# Unraveling the Gut–Liver–Brain Axis: Microbiome, Inflammation, and Emerging Therapeutic Approaches

**DOI:** 10.1155/mi/6733477

**Published:** 2025-06-18

**Authors:** Hiral Aghara, Manali Patel, Prashsti Chadha, Kirti Parwani, Ruchi Chaturvedi, Palash Mandal

**Affiliations:** P. D. Patel Institute of Applied Sciences, Charotar University of Science and Technology, Changa, Gujarat, India

## Abstract

The gut–liver–brain axis (GLB axis) plays a crucial role in maintaining metabolic, immune, and neurological homeostasis. The gut microbiota influences systemic health through its metabolites, including short-chain fatty acids (SCFAs), bile acids (BAs), and tryptophan (Trp) derivatives, which regulate immune function, lipid metabolism, and neurotransmitter balance. Dysbiosis is an imbalance in gut microbiota that has been implicated in metabolic dysfuntion associated fatty liver disease (MAFLD), alcohol-associated liver disease (AALD), and neuroinflammatory conditions such as schizophrenia. Increased gut permeability allows microbial byproducts like lipopolysaccharides (LPSs) to enter the liver and brain, activating inflammatory pathways that contribute to disease progression. Moreover, hepatic dysfunction can lead to neuroinflammation and cognitive impairments. Understanding the interplay between microbial metabolites and host physiology provides insight into novel therapeutic interventions. Strategies such as probiotics, prebiotics, synbiotics, fecal microbiota transfer (FMT), and postbiotics offer potential treatments to restore gut eubiosis and mitigate disease severity. This review highlights the mechanistic role of the GLB axis in health and disease, emphasizing microbiome-targeted therapies as a promising avenue for managing metabolic and neuropsychiatric disorders.

**Trial Registration:** ClinicalTrials.gov identifier: NCT04823676, NCT02496390, NCT06024681, NCT02721264

## 1. Introduction

The gut microbiota has emerged as a focal point of research due to its crucial role in maintaining overall health, leading to its designation as the “second brain.” Beyond its primary functions in digestion and nutrient absorption, the gut plays a vital role in xenobiotic and drug metabolism, immune defence, gut integrity maintenance, and the regulation of key organs such as the brain, liver, and kidneys [1–4]. A significant portion of these functions is mediated by the microbiome a highly diverse community of bacteria, fungi, and viruses that coevolved with humans to establish a complex symbiotic relationship [5]. The importance of gut health has been recognized for centuries. As early as 400B.C., Hippocrates famously stated, “Death sits in the bowels,” a notion that has evolved into the modern understanding of the microbiome, first formally described in the early 1900s [3, 5]. The gut serves as a vast interface between the host, environmental influences, and foreign antigens, housing a diverse microbial population collectively known as the gut microbiota. This microbiota is composed primarily of bacteria, archaea, and eukarya, making it one of the richest microbial ecosystems known on Earth. In the colon alone, microbial density reaches 10^12^ microbes per milliliter, contributing to an immense functional capacity of approximately 232 million genes, with metabolic functions comparable to the liver [6–8].

Large-scale studies such as MetaHit and the Human Microbiome Project have cataloged over 2172 microbial species from 12 different phyla inhabiting the gut, with 93.5% belonging to proteobacteria, firmicutes, actinobacteria, and bacteroidetes [9]. The balance between these microbial communities, termed eubiosis, plays a crucial role in gut integrity and immune homeostasis. A healthy gut microbiota, dominated by firmicutes and bacteroidetes, contributes to essential physiological processes such as insulin secretion, glucose tolerance, bile acid (BA) homeostasis, hepatic lipid storage, and leptin production in adipose tissue [4, 10]. Additionally, beneficial microbial byproducts, including short-chain fatty acids (SCFAs), succinate, and lactate, are critical for metabolic balance and overall health [11].

Gut microbiota is a dynamic microbial community that is influenced by age, gender, mode of birth, breast feeding, food intake, and environmental conditions, such as radiation and chemotherapy [6, 12–14]. Any perturbations in the gut microbial communities arising from external factors such as prolonged antibiotic treatment or introduction of a pathogen can result in dysbiosis that promotes the proliferation of harmful bacteria and diminishing beneficial strains. A dysbiotic microflora leads to an increased insulin resistance (IR), activation of inflammatory responses, and BA dysregulation [15]. Dysbiosis promotes the proliferation of harmful bacteria, triggering immune activation and compromising host homeostasis.

The gut microbiota plays a crucial role in maintaining overall health and homeostasis. This review focuses on gut microbes and their metabolites, particularly SCFAs and BAs, which contribute to anti-inflammatory responses. Dysregulation of these metabolites has been implicated in the progression of metabolism-associated liver diseases (MALDs), including metabolic dysfuntion associated fatty liver disease (MAFLD) and alcohol-associated liver disease (AALD). Additionally, gut-derived metabolites influence neurotransmission and neuroinflammation, linking gut microbiota to brain-associated disorders. Given the significant role of gut microbes and their metabolites in regulating metabolism, immune function, and disease progression, this review will explore their impact on liver and brain health. Furthermore, microbiome-based therapeutic approaches will be discussed as potential interventions for hepatic and neurological conditions.

## 2. Gut Microbiota and Their Role in Maintaining Gut Immunity

The gut microbiota provides multiple benefits, such as enhancing gut strength [16], offering protection against pathogens [17] shaping the intestinal epithelium, and regulating the immune system [18]. Its metabolites influence systemic immunity by moving from the gut lumen into circulation [19, 20]. Gut microbiota-derived metabolites, including SCFAs, tryptophan (Trp) metabolites, and BA metabolites, support the differentiation and function of immune-suppressive cells while suppressing inflammatory cell activity [21].

### 2.1. BAs

BAs are particularly significant due to their dual role as digestive agents and immune modulators, shaping both gut microbiota composition and host physiology. BAs are primarily synthesized in the liver from cholesterol and secreted into the small intestine as conjugated products. In the distal colon, gut microbiota transform them into secondary BAs, including lithocholic acid (LCA), deoxycholic acid (DCA), glycine, and taurine, which significantly influence immune responses in the colon [22]. BAs act as antimicrobial molecules, regulating gut microbiota composition by inhibiting the growth of certain microbes through ion gradient disruption, metabolite leakage, and bacterial membrane damage [23]. However, BA-tolerant microbes can thrive under these conditions, leading to gut dysbiosis and inflammatory diseases such as inflammatory bowel disease (IBD) [24, 25].

Beyond their antimicrobial function, BAs interact with macrophages, dendritic cells (DCs), regulatory T cells (Tregs), Th17 cells, intraepithelial lymphocytes, CD4 and CD8 cells, B cells, and natural killer (NK) T cells through nuclear and membrane receptors such as the farnesoid X receptor (FXR), G-protein-coupled BA receptor-1 (Gpbar-1/TGR5), and sphingosine-1-phosphate receptor 2 (S1PR2). These pathways regulate lipid, glucose, and energy metabolism and are crucial for maintaining gut and systemic homeostasis [26, 27]. Among these, FXR signaling plays a key role in metabolic regulation across the liver, intestine, and adipose tissues [28]. Additionally, gut microbiota particularly bacteroidetes play a major role in BA metabolism. Transcriptomic analysis identified bacteroidetes enzymes capable of converting 3-oxo-LCA (3-oxoLCA) into iso-allo-LCA (isoalloLCA), which inhibits the growth of non-bacteroidetes species while modulating immune responses. IsoalloLCA further promotes nTreg cell differentiation via NR4A1-binding sites, activating Foxp3 transcription and enhancing immune tolerance [29].

Thus, BAs are not only digestive agents but also key regulators of gut microbiota and immune function, influencing both intestinal and systemic health.

### 2.2. SCFAs

Gut microbiota-derived metabolites, particularly SCFAs, succinate, and lactate, play a crucial role in maintaining metabolic balance and intestinal homeostasis [11]. SCFAs, primarily acetate, propionate, and butyrate, are key products of dietary fiber fermentation by gut microbes, with Clostridium IVa and XIVa being dominant butyrate producers and bacteroidetes spp. primarily generating propionate [22, 30, 31]. These microbial metabolites are central to immune regulation, displaying anti-inflammatory properties and modulating immune cell function [32].

SCFAs exert their immunomodulatory effect majorly by activating G-protein-coupled receptors (GPCRs) GPR109A, GPR41, and GPR43. Butyrate has the highest affinity for all three receptors, whereas acetate and propionate selectively activate GPR41 and GPR109A [33]. Notably, GPR109A is expressed on immune cells such as macrophages, DCs, and neutrophils, where its activation suppresses pro-inflammatory cytokine production. Similarly, GPR41 and GPR43, expressed on monocytes, DCs, eosinophils, and neutrophils, contribute to a systemic anti-inflammatory response. Additionally, SCFAs regulate immune function via monocarboxylate transporters (MCTs) and histone deacetylase (HDAC) inhibition, further influencing macrophage polarization and immune tolerance [34].

SCFAs are also crucial for Treg development, particularly RORγt + FoxP3 + pTregs, which predominate in the colon and are significantly reduced in germ-free mice [35, 36]. Butyrate, specifically, enhances peripheral CD4 + T-cell differentiation by inhibiting HDACs, leading to Foxp3 promoter acetylation and Treg differentiation [30, 37]. In addition to SCFAs, retinoic acid also plays a role in RORγt + Treg differentiation, independent of microbial influence. DCs further facilitate Treg differentiation either by suppressing RelB and pro-inflammatory gene expression or through receptors such as GPR109A, butyrate, and vitamin B3 [38].

**Figure 1:**
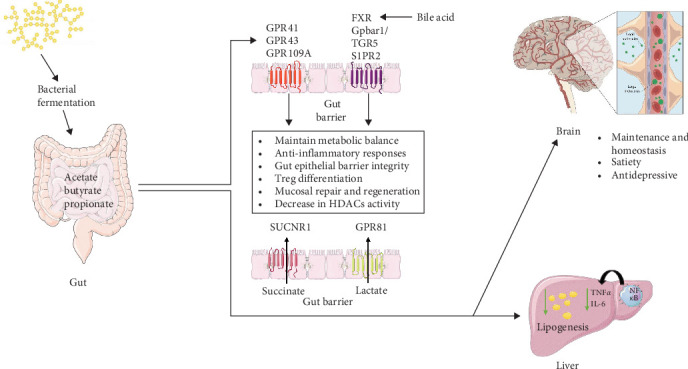
Role of SCFA in maintaining gut–liver and gut–brain axis. SCFAs, bile acids, and other metabolites such as lactate and succinate play a crucial role in maintaining homeostasis through receptor-mediated signaling. SCFAs, produced via bacterial fermentation, interact with G-protein-coupled receptors (GPR41, GPR43, and GPR109A) to regulate metabolic balance, anti-inflammatory responses, and gut epithelial barrier integrity. Additionally, other signaling molecules, including bile acids, succinate, and lactate, influence metabolic regulation through specific receptors such as FXR, TGR5, Gpbar1, and S1PR2, which are expressed by key immune cells. These interactions contribute to gut–liver and gut–brain communication, impacting processes such as lipogenesis, immune modulation, and neuroprotection.

Overall, SCFAs and other gut microbiota-derived metabolites serve as crucial modulators of immune function, inflammation, and metabolic homeostasis, highlighting their significance in maintaining gut and systemic health which is explained in Figure 1.

### 2.3. Gut Microbiota and Immune System

Given the enormous microbial inhabitants of the gut, the host has evolved a robust immune surveillance system to confine microbial symbionts to their niche while also restricting the translocation of microbes and their metabolites. The intestinal barrier plays a critical role in this process and is primarily divided into two sections: the mechanical barrier and the chemical barrier. The mechanical barrier consists of epithelial cells, a lipid layer, and tight junctions, forming the first line of defence. Beneath this, the chemical barrier often referred to as the “Mucosal Firewall” is composed of a mucus layer produced by goblet cells (GCS), antimicrobial peptides, and secretory IgA (sIgA), all of which contribute to gut homeostasis [39]. Beyond its physical barrier function, the gut microbiota actively interacts with the immune system, playing a crucial role in immune cell maturation and activation [40] further facilitating:

• Mucin secretion by GCs, ensuring barrier integrity.
• Development of intestinal mucosa-associated lymphoid tissue (MALT) to enhance immune defence.
• Immune cell differentiation and maturation, particularly through the stimulation of isolated lymphoid follicle development and the activation of naïve T and B cells [41].

The gut microbiota is separated from host cells by a mucosal epithelium formed by tightly connected intestinal epithelial cells (IECs), which serve as a physical and chemical barrier [42]. Tissue-resident CD4T, CD8T, and innate lymphoid cells (ICLs) release cytokines like interleukin (IL)-17 and IL-22, crucial for maintaining intestinal barrier function. A disruption in this regulation can damage the intestinal barrier and cause inflammation [43].

The gut microbiota has an important role in shaping the mucosal firewall, such as and RegTIIIγ, a gram positive bacteria specific antimicrobial peptide, are secreted in MyD88 dependent manner in response to the presence of commensal [44]. Similarly, sIgA is produced in response to the presence of antigen presenting cells (APCs) from the lamina propria (LP). B-cells in the mesenteric lymph nodes produce sIgA in response to APCs from the LP. The sIgA then transcytosed through the epithelial cells and contribute towards restricting the microbiota [44]. Intestinal DCs within the cytokine and chemokine rich environment of Peyer's patch aid in B-cells class switching from IgM producing B cells to IgA producing cells [45]. Group 3 ICLs (ICL3s), found abundantly in the LP of gut, represent an arm of the innate immunity. ILC3s have also been proven to require the help of DCs, macrophages, monocytes to establish connections with microbiota. The major transcription factor produced by the ILC3s are RORγt and Ahr [46]. Their development is driven by IL-23 and IL-1β produced by APCs, in presence of pathogens such as *Citrobacter rodentiumI* which promotes IL-22 production by ILC3s in a MyD88 signaling dependent pathway leading to the enrichment of GM-CSF is mainly produced by RORγt + ILC3s, can stimulate the release of IL-10 and other cytokines from DCs and monocytes to promote Treg differentiation and suppress Th17 cells [46–48]. IL-22 the produced by ILC3s promotes differentiation of mucous producing GCs and IECs from crypt stem cells [49, 50]. IL-22 leads to the secretion antibacterial peptides by Paneth cells and IECs reinforcing the gut barrier [46, 51].

sIgA from LP provides transient protection against the commensals as they lack classical memory. T-cells of the gastric mucosa play an important part in providing long term protection and in developing an immune tolerant environment of the gut. Of the T-cells that provide homeostatic environment include Th17 cells that produce IL-17 and IL-22 that contribute to the homeostatic dialog. The differentiation of naïve T-cells is influenced by the presence of microbiota, their products as LPS [44] bacterial metabolites as SCFA [30] and indole derivatives [52]. Combined effect of Th17/Treg creates an immune tolerant environment of the gut. Kim et al. [35] demonstrated that the peripheral Th17 cells contribute to autoimmunity and lead to neuro-inflammation, while the colonic Th17 help to develop immune tolerant environment. In LP, Th17 develops from either naïve T-cells or from FOXP3 + Treg cells under the influence IL-6, IL-22, and TGF-β [53]. On the other hand, Treg cells develop in an immune environment enriched in TGF-β that lack Il-6 and IL-22. This development of Tregs and Th17 is dynamic, wherein trans differentiation of Treg and Th17 is driven by appropriate metabolic and chemokine milieu [35, 52–54]. A homeostatic gut environment is skewed in favor of anti-inflammatory T-cells, while pro-inflammatory subsets of T-cells, Th1 and Th2, are greatly inhibited. Th17/Treg axis develops in response to symbiont and its metabolites. One of the key inducers of the Th17 have been demonstrated to be segmented filamentous bacteria (SFB), a bacteria known to have the ability of inhabit the “demilitarized zone,” attached to the epithelial gut cells, of the mucosal firewall [39, 55, 56]. Similarly, polysaccharide A (PSA) from the human commensal *Bacteroides fragilis* promotes IL-10 production by intestinal FoxP3 + Treg cells via toll-like receptor (TLR)-2-dependent manner [38].

**Figure 2:**
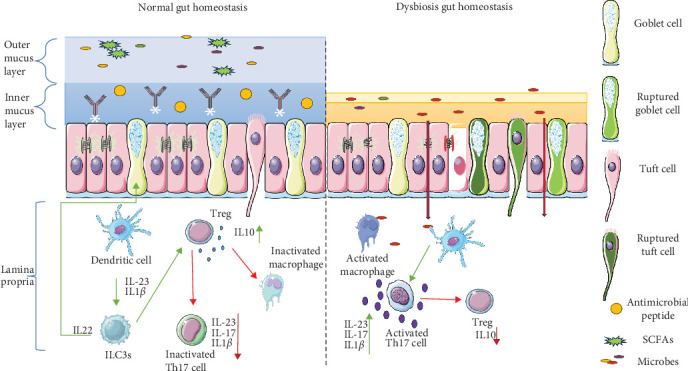
Gut homeostasis. In normal condition SCFAs and other intestinal cells help to maintain the immunity by regulating the T cell differentiation. In normal condition goblet cell produce mucus layer which is dense in nature and contains several antimicrobial peptide and immunoglobulins. Further, the tight junction proteins like occludin, zona occludin-1/2/3, and claudin help to maintain the barrier integrity. While in infection or in dysbiotic condition mucus layer is ruptured goblet cells are impaired and tuft cells become hyperactivated, contributing to an inflammatory response. Tight junction integrity is compromised, increasing gut permeability. DCs in this condition activate Th17 cells, leading to an upregulation of proinflammatory cytokines (IL-23, IL-17, and IL-1β), further exacerbating inflammation. The balance between immune regulation and inflammation is disturbed, promoting gut barrier dysfunction and disease progression.

Th17 cells in vivo depend on enhanced cholesterol biosynthesis, glutaminolysis pathway, and increased OXPHOS for cytokine production whereas peripheral Th17 cells are enriched in genes related to the glycolytic pathway [57]. Th17 that develop under homeostatic conditions such as in presence of SFB downregulates glycolysis and rely on OXPHOS for secretion of cytokines such as IL-10 to promote Treg development [58]. In contrast Th17 developing in presence of pathogens have enhanced glycolytic pathways, amino acid metabolism, and purine metabolism that fuels the production IL-17 and interferon-γ (IFN-γ) [57, 59].  Figure 2 elaborates on the mechanism that maintains gut homeostasis and illustrates how its disruption or malfunction can lead to inflammation and gut dysbiosis.

### 2.4. Mucus Layer

There is another chemical layer generated via epithelial cells known as mucus layer which is divided into three layers according to their activity. First is lumen, second layer is composed of microbes and their byproducts, while the third one near to the mechanical barrier is composed of antimicrobial proteins and immunoglobulin [60]. This mucin layer is formed by GCs which forms a disulfide bounded hydrogel overlaying on epithelial cells [61, 62]. In the colonic mucus layer, muc2 heavily glycosylated. Mucus also acts as an adhesive molecule for bacterial colonization. Approximately 30% of gut commensals express type IV pili, long and thin surface appendages that facilitate bacterial adhesion but are involved in motility, DNA exchange, and protein uptake [63]. The mucus layer is consisted of many anti-inflammatory and antimicrobial peptides which act as a protective shield and prevent bacterial translocation [64–66]. Moreover, to prevent bacterial penetration, the inner layer is replaced with the new one at every 1–2 h [64]. Along with that antimicrobial molecule get mixed with mucus layer and help in generating immune response.

In correspond to these mechanism gut provides the immunity and maintain the homeostasis. As mentioned, these mechanisms can be sabotaged by other factors and might rise as a diseased condition. These can happen if gut microbial activity or components are changed which is normally known as gut dysbiosis. As gut dysbiosis take place it would majorly affect the liver and brain and cause disease.

## 3. Disease Related to Microbial Change and Effect With GLB Axis

The GLB axis is essential for maintaining homeostasis by regulating immune, metabolic, and neurological activities. The liver, being the body's largest immune organ, generates metabolites that circulate through the bloodstream to the gut and brain, impacting immune regulation and inflammation. Under healthy conditions, these organs function synergistically; however, during disease, this balance is disrupted, leading to immune dysfunction. Recent studies emphasize the gut microbiota's significant role in influencing neurological and social well-being [67, 68], highlighting the deep interconnection among these organs. For instance, severe liver conditions like cirrhosis are frequently linked to hepatic encephalopathy (HE), a disorder that compromises neuronal integrity. Additionally, alcohol-related liver failure can cause the accumulation of neurotoxins such as ammonia and manganese, leading to impaired brain function. Dysbiosis of the gut microbiota can further aggravate both liver and brain dysfunction through the production of harmful microbial metabolites and toxic byproducts.

### 3.1. Steatohepatitis

Liver diseases are increasingly becoming major global health concerns. Due to unhealthy diet and sedentary life style it causes liver disease is described in the setting of MAFLD as well as AALD. Although both conditions involve fat accumulation in the liver, their underlying mechanisms differ, yet both significantly contribute to the rising burden of liver diseases worldwide [69], leading to obesity and related complications. The meta-analysis by Cheemerla and Balakrishnan [70] indicated that 27.3% deaths in men and 20.6% deaths in women are due to cirrhosis by AALD, whereas, deaths due to cirrhosis caused by MAFLD is 7.7% for men and 11.3% for women. According to World Health Organization (WHO), the average daily alcohol consumption of more than 60 g for men and 40 g for women result in high risk of AALD. Further, MAFLD is caused by the prevalence of unhealthy diet, sedentary lifestyle has led to serious health consequences in terms of obesity and associated problems [71]. Despite their different causes, both AALD and MAFLD share similar pathological spectra, ranging from simple fat accumulation (steatosis) to hepatitis to cirrhosis, and eventually hepatocellular carcinoma (HCC) [72].

### 3.2. AALD

AALD encompasses a range of liver conditions primarily caused by excessive alcohol intake. As alcohol consumption increases, ethanol is metabolized into acetaldehyde by alcohol dehydrogenase (ADH), and subsequently into acetate by acetaldehyde dehydrogenase (ALDH) [73, 74]. However, chronic alcohol intake leads to the accumulation of acetaldehyde, which forms DNA and protein adducts, contributing to cellular damage. Prolonged exposure of alcohol or acetaldehyde can also mutate ALDH function, further lowering acetate levels and disrupting liver metabolism by forming acetaldehyde adducts. This cascade of effects not only damages the liver but also affects the GLB axis, highlighting the systemic impact of long-term alcohol consumption [75].

AALD is an umbrella term encompassing conditions such as alcohol-associated steatosis (AAS), steatohepatitis (ASH), liver fibrosis, cirrhosis, and HCC [76]. Disease progression involves multiple mechanisms at each stage. Early stages may be reversible with lifestyle changes and abstinence, but continued binge drinking advances the condition to irreversible stages. Excessive alcohol intake activates oxidative pathways involving CYP2E1 and catalase, leading to ROS production and cellular damage [77, 78]. Elevated CYP2E1 activity also induces early growth response genes and increases sterol regulatory element-binding protein 1c (SREBP1c) transcription, promoting de novo lipogenesis via acetyl-CoA carboxylase (ACC) and fatty acid synthase (FAS) [79–81]. This lipogenesis contributes to inflammation, inhibits autophagy through mTORC activation, and enhances pro-inflammatory signaling via STAT1, tumor necrosis factor-alpha (TNF-α), and NF-κB, while reducing β-oxidation and promoting fat accumulation [80, 82, 83]. Additionally, gut dysbiosis can exacerbate inflammation and liver damage.

In healthy conditions, a functional intestinal barrier prevents intact bacteria from entering the liver, despite the direct connection between the gut and the liver via the portal vein. However, bacterial genetic products (mRNA) and lipopolysaccharides (LPS) are detectable in portal blood and liver, indicating that gut-derived bacterial components may influence liver function in normal physiology [84]. Excess amount of alcohol would cause liver damage primarily but along with that it would also damage the intestinal barrier and change the composition of gut flora and causes gut dysbiosis. Research suggests that gut dysbiosis as a potential contributor of fatty liver diseases. Alcohol consumption has been found to alter the gut microbiota by increasing the abundance of proteobacteria, enterobacteriacea, and streptococcus and decrease the abundance of bacteroides, akkermansia, and faecalibacterium [85] and showed increased proteobacteria and decreased in bacteroidetes observed, which further is responsible for leaky gut condition [86]. Some studies reported an increase in proteobacteria and a decrease in bacteroidetes [87, 88]. Alog with that in alcoholics, increase of firmicutes like *Enterococcus* spp. and decrease in *Akkermansia muciniphila* (phylum Verrucomicrobia) is also observed. *Akkermansia muciniphila* tends to protect the liver against the alcohol injury by producing ornithine and decreased production of oxalic acid which further beneficial for gut health by increasing the production of mucus layer [89, 90]. Due to excessive ethanol microRNA212 activity would interfere with tight junction protein activity like ZO-1 and disrupts it [73, 91]. Eventually, TLR-4 on hepatic stellate cells (HSCs) would also get triggered due to LPS and results in expression of IL-6, TGF-β1, and monocyte chemotactic protein (MCP-1). Even, HSC cells would also get activated via MyD88 mechanism which causes inflammation and also decreases the expression of miRNA 29 which further results in extracellular matrix production [92, 93].

Individuals with alcohol use disorder (AUD) often exhibit anxiety and depression, which may be linked to changes in intestinal microflora. The gut–brain axis, involving various neurotransmitters, hormones, and metabolites, plays a crucial role in maintaining homeostasis. Alterations in gut microflora can affect the secretion of key neurotransmitters such as GABA, serotonin, and dopamine, exacerbating symptoms of depression and anxiety.

Microbial products like SCFAs and GABA can act as neurotransmitters, influencing both peripheral and central nervous systems, thereby modifying host mechanisms [94]. These microbial metabolites can travel through systemic circulation, cross the blood–brain barrier (BBB), and potentially induce inflammation or other neural dysfunctions. Treatment with Pueraria extract has been shown to modify gut microbial composition, increasing the abundance of *Bacteroides*, *Ruminococcus*, and *Prevotella*. This results in the production of SCFAs, which protect the intestinal wall. The bidirectional interaction between the gut and brain means stress can alter gut microflora, leading to increased intestinal permeability [94, 95]. Gut dysbiosis in individuals with AUD is associated with inflammation, partly through the activation of peripheral blood mononuclear cells [96]. Changes in gut microflora and metabolites can increase GABA production, which may contribute to neuronal damage and alter vagus nerve signaling, further intensifying anxiety [97, 98]. Additionally, heightened activity in the cortex and hippocampus can increase BBB permeability, resulting in neuronal loss within the hippocampus and the generation of neurotoxic effects like necrosis [99, 100].

As excessive amount of alcohol contribute in damaging GLB axis majorly affecting liver by further increasing the damage via gut. Further, liver would also get affected via sedentary life style causing MALD.

### 3.3. MALD

MAFLD is characterized by abnormal fat accumulation in hepatocytes, independent of significant alcohol intake, and is closely linked with features of metabolic syndrome, including IR, type 2 diabetes, and obesity [101]. MAFLD is classified into two distinct types: simple steatosis and metabolism associated steatohepatitis (MASH) [102]. The pathogenesis follows the “two-hit” hypothesis: the first hit involves increased lipid accumulation due to excessive caloric intake and upregulated de novo lipogenesis [103–105], while the second hit includes inflammation, oxidative stress, and lipid peroxidation, which together contribute to disease progression [106]. Lipotoxicity impairs the functional activity of hepatocytes and adipose tissue.

Steatosis arises due to excess calorie intake, leading to accumulation of triglycerides, sphingolipids, and phospholipids in hepatocytes [107]. Free fatty acids (FFAs) that contribute to hepatic triglycerides formation primarily originate from three sources: fat and de novo lipogenesis: 59% of FFAs come from adipose tissue lipolysis, 26% from de novo lipogenesis, and 15% diet [108, 109]. Notably, studies have shown that in MAFLD, enhanced hepatic de novo lipogenesis and elevated secretion of very low-density lipoprotein (VLDL) appear to be compensatory responses to the increased influx of fatty acids into the liver [110].

MAFLD represents the initial stage of MALD and is primarily caused by lifestyle factors and an improper diet. It is strongly associated with obesity and IR, such as in type 2 diabetes mellitus. IR increases FFA concentrations in the liver, which upregulates leptin levels and downregulates adiponectin activity in adipose tissue. This imbalance results in increased fat accumulation, while adipose tissue fails to synthesize lipids effectively, leading to hepatic steatosis [111]. Excessive lipid production disrupts endoplasmic reticulum function, exacerbating the condition. This disruption induces inflammation, oxidative stress, and subsequent cell death.

In MAFLD, lipotoxicity is further driven by the activation of SREBP1c. Enhanced SREBP1c activation promotes inflammation by upregulating TNF-α [112]. Additionally, FAS and ACC contribute to inflammation by acting as lipotoxic factors [107]. As the disease progresses, the dysregulation of Th17 and Treg cells exacerbates inflammation, leading to the production of IL-6 and TNF-α. This results in elevated IL-17 activity, further advancing the disease. IL-17 promotes neutrophil infiltration and the production of pro-inflammatory cytokines and chemokines, thereby aggravating the inflammatory response [113, 114].

The close connection between the gut and liver underscores the significant regulatory effect of the gut microbiota on liver health. MAFLD patient tends to show higher abundance gram negative bacteria then gram positive bacteria and showed higher abundance of proteobacteria [115, 116]. Disruptions in the intestinal microbiota, often induced by diets with high saturated fats and fructose, can increase intestinal permeability and contribute to chronic inflammation [117]. Remarkably, a continuous low-grade inflammatory state may result in an acceleration in the progression from hepatic steatosis to MASH [118]. Hepatic steatosis is commonly observed in patients with obesity and diabetes and animals model subjects fed with a high-fat diet (HFD). The regulation of hepatic lipid metabolism is a multifaceted process, involving fatty acid synthesis, uptake, oxidation, and release which are basic regulatory mechanisms for fat accumulation in the liver [119]. A study aimed at exploring the relationship between lipid metabolism found that rats fed a HFD are treated with antibiotics (abs) to induced dysbiosis exhibited significantly higher levels of HDL, LDL, total cholesterol, and triglycerides, as compared to both the HFD and control groups. Additionally, a distinct shift in the gut microbiota composition was observed in the HFD + ab group [120]. These findings indicate that alteration in gut microbiota composition can elevate serum lipid levels, potentially leading to liver deposition of fats and evolution to steatohepatitis. Gut derived metabolites can cause capillarisation of liver sinusoidal endothelial cells (LSECs) and participate in liver inflammation [121]. Activation of LSECs cause liver inflammation by increased activity of IL6, TNF-α, and CCL2 [122]. Gut derived metabolites like SCFAs, trimethylamine-N-oxide (TMAO), endogenous ethanol, and other metabolites leads to alteration of intestinal barrier function and intestinal immune system [115]. SCFAs ameliorate NAFLD, while TMAO can elevate the MAFLD. TMAO is metabolite of choline which is generated from gut microbes can influence BA synthesis and cause IR [123].

The liver–brain axis is another important factor influencing fat accumulation in the liver. The autonomic nervous system has been shown to interact with the liver. Research on rat liver model has highlighted a relationship between increased hepatic fat mass aging and the stimulation of adenylyl cyclase activity by isoproterenol and agonist of the beta-adrenergic receptor [124]. The administration of isoproterenol to young and old rodent in vivo increases liver fat accumulation and sympathetic nerve activity increases in mice fed a HFD [125]. Fatty liver diseases can adversely affect the GLB axis by causing inflammation by Kupffer cells activation due to endotoxins which are primarily secreted in the gut. This would further cause neuronal inflammation and cause damage to circadian rhythm. Additionally, the gut microbiota is closely linked to GLP-1 via GPR41/43 pathway, contributing to fat accumulation and the development of fatty liver disease [126]. Gut dysbiosis further enhances DCA activity, leading to microglial activation in the brain and the activation of the HPGD/JAK2/STAT3 pathway [127].

Gut microbiota dysbiosis and its byproducts play a crucial role in the progression of both types of fatty liver disease and the activation of inflammatory pathways, which will be further discussed in the next section. In summary, an imbalanced gut microbiome produces toxic metabolites that compromise the intestinal barrier, allowing harmful substances to enter the liver via the portal vein and the brain through the BBB. In the liver, these toxins target Kupffer cells, driving M2 to M1 polarization, while in the brain, they trigger neuroinflammation by activating microglia and astrocytes.

## 4. Gut Dysbiosis and Its Impact on M1 and M2 Polarization in Liver

As discussed in Section 2.3, the gut microbiota along with maintaining immunity it also plays a pivotal role in lipid metabolism, reducing fat accumulation, and lipid storage, while enhancing leptin production in adipose tissue. Diet and life style causes changes in bacterial populations from beneficial microbes like *Bacteroidetes*, *Ruminococcus*, and *Roseburia* to pathogenic gram negative bacteria such as *Fusobacteria*, *Proteobacteria*, and *Streptococcaceae* [128, 129]. This shift reduces the presence of SCFA producing bacteria, further contributing to gut leakiness, systemic inflammation, and neuroinflammation through endotoxins like LPS [130, 131].

Due to gut dysbiosis, microbial metabolites like LPS is released from the leaky gut enters the liver via the portal vein and activates TLR-4, a key player in alcohol-related liver inflammation. Both chronic and acute alcohol consumption can increase TLR-4 expression. LPS binds with CD14 and LPS-binding protein (LBP) to form a complex that activates TLR-4, triggering liver macrophages (Kupffer cells). Activated Kupffer cells induce transcription factors like NF-κβ, leading to the production of pro-inflammatory cytokines (e.g., TNF-α, IL-6, and IL-1), chemokines, and reactive oxygen species (ROS). LPS also activates HSCs and MyD88-dependent pathways, producing inflammatory cytokines and TGF-β [76].

Along with that, leaky gut is often triggered by factors like alcohol consumption and HFD or high-fructose diet, which increases the release of endotoxins such as LPS. High ethanol intake elevates bacterial ALDH activity, disrupting the tight junctions between intestinal cells. Tight junctions rely on proteins such as claudins and occludin, which interconnects with junctional membrane proteins like Zonula Occludin (ZO)-1/2/3. Patel et al. [132] observed that ethanol-induced tight junction damage reduced the expression of ZO-1 and occludin in Caco-2 cell monolayers. Furthermore, NK cells and NKT cells also interact with gut microbiota and may influence liver disease progression. NK cells bridge innate and adaptive immunity and exhibit antifibrotic effects [133]. Meanwhile, NKT cells, which share features of both NK and T cells, secrete cytokines and regulate immunity by producing pro-inflammatory Th1 and anti-inflammatory Th2 cells [134]. Overall, gut microbiota-induced hepatic cell activation and leaky gut-related endotoxemia are key contributors to liver disease pathogenesis.

In liver disease, macrophage polarization and the switch between M1 and M2 phenotypes are primarily driven by environmental cues and signaling pathways. The TLR4/MYD88 pathway is a classical mechanism that increases inflammation by activating M1 macrophages. This activation is triggered by gut-derived LPS, which stimulates Kupffer cells (resident liver macrophages) and HSCs, thereby amplifying liver injury and fibrosis progression [135, 136].

Kupffer cells oscillate between M1 (pro-inflammatory) and M2 (anti-inflammatory) phenotypes. LPS favors the M1 state, exacerbating liver inflammation in alcohol-induced liver disease [137]. The switch between M1 and M2 polarization is modulated by multiple factors, including gut metabolites, cytokines, and signaling molecules like IFN-γ, IL-4, IL10, HO-1, and arginase activity [138]. For instance, LPS promotes M1 polarization, whereas SCFA like butyrate enhance STAT6 activation via IL-4 secretion, favoring M2 polarization [139]. IFN-γ and TNF-α drive M1 polarization through the NF-κB and STAT1 pathways, while IL-10, IL-4, and PPARγ [140] activation promote M2 polarization by upregulating STAT3 and STAT6. Additionally, arginase 1 secretion and HO-1 activity play roles in reducing inflammation and supporting M2 polarization [141].

M1 macrophage polarization is associated with increased TNF-α and NF-κB activity, leading to elevated inflammation and reduced STAT6 signaling. In contrast, M2 macrophages rely on STAT3 and STAT6 upregulation to maintain an anti-inflammatory environment in the liver [142]. The JAK/STAT pathway, a key regulator of macrophage function, is influenced by the TLR4/NF-κB axis. TLR4 activation enhances STAT1 expression, promoting M1 polarization, while simultaneously suppressing STAT3 and STAT6, thus, limiting M2 polarization [143–145]. M1 macrophage polarization would increase due to increased activity of TNF-α and NF-κB and increases the inflammation rate and reducing STAT6 activity which can be improvised by PPARγ activation that helps in lipogenesis [146]. Ren et al. [147] demonstrated in a murine model that M2 macrophage activity was increased, as evidenced by elevated markers like Arg1, while M1 markers like iNOS were decreased. These findings highlight the dynamic interplay between macrophages, gut metabolites, and liver-resident cells in modulating the progression of liver disease.

Emerging research suggests that reduced intestinal barrier integrity contributes to the progression of MAFLD. Obese individuals with MAFLD show increased gram negative bacteria levels, resulting in significant endotoxemia compared to healthy individuals [148]. LPS activates TLR-4 pathways in endothelial cells and TLR-9 in DCs, causing chemokine release (e.g., CCL2, CXCL2, CXCL10, and CXCL16) and liver damage [149].

The brain is closely connected to the liver and gut through proinflammatory signaling, which decreases the activity of tight junction proteins in the gut, leading to increased gut permeability [150, 151]. Fatty liver disease also affects several neuronal pathways, one of which is the activation of the sympathetic nervous system, further disrupting the intestinal barrier. When LPS reach the brain, they trigger inflammation and activate the hypothalamic–pituitary–adrenal (HPA) axis, impacting brain function. Increased HPA axis activity leads to the release of adrenocorticotropic hormone, which exacerbates intestinal permeability [94, 150].

**Figure 3:**
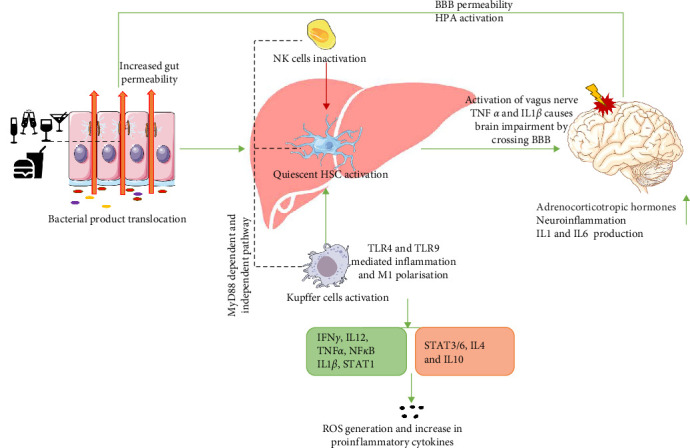
Gut–liver–brain axis disrupted homeostasis. Gut dysbiosis, often triggered by excessive alcohol consumption or a high-fat diet, disrupts tight junction proteins in the intestinal lining, allowing microbial byproducts particularly endotoxins such as LPS to translocate to the liver via the portal vein and to the brain via the vagus nerve. Stress-induced gut inflammation further exacerbates this disruption by increasing inflammatory markers, enhancing blood–brain barrier (BBB) permeability and promoting neuroinflammation. In the liver, LPS activates pattern recognition receptors like toll-like receptor 4 (TLR-4) on Kupffer cells and hepatic stellate cells HSCs, initiating a pro-inflammatory cascade. This leads to M1 polarization of Kupffer cells via pathogen-associated molecular patterns (PAMPs) and the suppression of M2 macrophages, which are normally anti-inflammatory. The shift from M2 to M1 polarization increases the secretion of inflammatory cytokines and chemokines, including TNF-α and activation of the NF-κB pathway. Additionally, LPS-induced TLR-4 activation enhances STAT1 expression, further amplifying inflammation, while elevated TNF-α levels inhibit STAT6, a key anti-inflammatory regulator. These inflammatory signals not only worsen liver pathology by stimulating further HSC activation and fibrosis but also extend to the brain, where LPS-induced inflammation triggers the release of adrenocorticotropic hormone. This hormonal response feeds back into the gut, contributing to additional tight junction disruption and perpetuating the cycle of gut–liver–brain axis dysfunction.

Gut dysbiosis significantly impacts the liver, which in turn can lead to neuroinflammation by activating astrocytes and microglia. This activation may result from gut derived products crossing the BBB or from liver derived factors, as explained in Figure 3.

## 5. Gut Dysbiosis and Neuroinflammation

Gut barrier and BBB are closely connected through vagus nerve. There is bidirectional communication between gut to brain by neurotransmitters and gut metabolites. Gut metabolites like SCFAs, BAs, and succinate and few other amino acids. The major connection with gut intestinal tract and central nervous system is connected through vagus nerve and also help in signaling. The gut, liver, and brain are interconnected through multiple pathways, including microbiota derived metabolites (SCFA, neurotransmitters, and inflammatory molecules), immune system activation (proinflammatory cytokines affecting brain function), neurotransmitter regulation (serotonin, dopamine, and glutamate balance), and the vagus nerve (direct gut–brain signaling) [152, 153]. Recent studies suggest that gut brain axis plays a critical role in neuropsychiatric disorders such as depression, anxiety including schizophrenia and even in neuroinflammatory diseases like HE. In a dysbiotic state, gut metabolites and bacteria can cross the BBB, leading to its disruption and triggering systemic inflammation. This can result in neuronal cell death and demyelination. Additionally, dysbiosis promotes Th17 cell proliferation in the gut due to increased ROS generated by SFB. Mucin degradation further activates microglia and astrocytes, exacerbating inflammation [154].

The GLB axis is closely connected in schizophrenia. There would be change in gut microbes like increased abundance of *Escherichia coli* decreased bifidobacteria can cause decreased, which further decreases the GABA and 5-HT along with increased inflammatory cytokine release. Gut dysbiosis can alter the neurotransmitter production as well like certain *Lactobacillus* and *Bifidobacterium species* produce gamma aminobutyric acid; *Escherichia*, *Bacillus*, and *Saccharomyces sp*. produce noradrenaline; *Streptococcus*, *Escherichia*, and *Enterococcus sp*. produce 5-HT; *Bacillus* produces dopamine; and *Lactobacillus* produces acetylcholine [155–157]. It causes the gut barrier alteration and causes BBB alteration. Due to BBB disruption proinflammatory cytokines are released causing neuroinflammation in brain with activated microglia and astrocytes [158, 159]. Transplantation of *Streptococcus vestibularis*, in mice caused development of schizophrenia-like behavior [160]. In schizophrenia patient, there is decreased abundance of *Faecalibacterium*, *Roseburia*, and *Butyricicoccus*, which play a role in butyrate production [161]. Concurrent with this it has been demonstrated that patients suffering from schizophrenia have lower α and β diversity indices [162, 163]. Schizophrenia patients also demonstrate translocation of members of oral microflora as member of the genera *Streptococcus* and *Actinomyces* with concomitant decline of the microbial genera associated with the butyrate producers as *Roseburia*, *Faecalibacterium*, *Coprococcus*, *Anaerostipes*, and *Fusicatenibacter* in the gut [162]. Absence of key genera involved in the butyrate breaches the gut integrity and result in inflammation as butyrate act as anti-inflammatory metabolite that induces the production of IL-10, IL-18, and favors the development of Treg cells by inhibiting the HDACs [30], HDACs serve as major chromatin remodeler that are known to be present at elevated levels in the frontal cortex, hippocampus, and medial temporal lobe of schizophrenia patients [164]. Gut microbiota disturbances can also promote low-grade systemic inflammation, leading to BBB dysfunction and inflammatory responses in schizophrenia and elevated pro-inflammatory cytokines, such as IL-6/4/1β/8 and TNF-α, suggest that gut-driven immune activation plays a role in psychiatric symptoms [161, 165, 166]. Along with that Trp metabolism and increased production of serotonin and dopamine also gained focus by many researchers due to its complex dysregulation mechanism and inflammation [167, 168].

The liver plays a crucial role in maintaining metabolic balance and detoxification, both of which influence brain function. Liver dysfunction can contribute to neuroinflammation, a hallmark of schizophrenia, through increased inflammation and oxidative stress. Additionally, impaired toxin metabolism may lead to an accumulation of neurotoxic substances that negatively impact cognitive function. Furthermore, disruptions in lipid metabolism and energy regulation as indicated by altered lipid profiles in individuals with schizophrenia suggest that metabolic imbalances may be linked to the disorder [169]. Schizophrenia is frequently associated with metabolic syndrome, IR, and liver dysfunction, which may result from gut-derived toxins disrupting liver metabolism. One such condition, MAFLD is commonly observed in schizophrenia patients and is linked to systemic inflammation and insulin dysregulation, which, in turn, impact brain function. Impaired liver function can reduce the body's ability to clear neurotoxic substances, leading to oxidative stress and further neuroinflammation [170–172]. Stiernborg et al. [163] revealed that *A. muciniphila* was one of the microbial populations negatively affected in schizophrenia patients as compared to the control. Numerous studies have demonstrated the beneficial role *A. muciniphila* regulating metabolism in the host, and therefore, a lack of *A. muciniphila* is associated with obesity [173]. Due to its impact in regulating obesity, *A. muciniphila* is forwarded as probiotic to relieve MAFLD. Rao et al. [174], in mice model, demonstrated that *A. muciniphila* in HFD fed obese mice relieves MAFLD condition by reducing inflammation and promoting mitochondrial oxidation, improving BAs metabolism and reshaping the gut microbiome by reducing apoptosis induced inflammation in the gut. These two independent studies by Rao et al. [174] and Stiernborg et al. [163] reaffirm a possible role of GLB axis in development of psychotic conditions as schizophrenia. The GLB axis presents a fresh perspective on the underlying mechanisms of schizophrenia. Further studies are essential to refine microbiome and liver focused therapies as complementary treatments for this disorder.

Along with the schizophrenia, HE is also one of the neuropsychiatric and neuroinflammatory condition which is reversible that involves liver failure and leaky gut [175, 131]. It has a wide spectrum of symptoms, ranging from lack of awareness and altered sleep patterns in the early stages, to personality changes and incomprehensible speech in the intermediate stage, and finally coma in the end stage [175]. The development of HE can be attributed to various factors, one of which is hyperammonemia [176]. Ammonia detoxification primarily occurs in the liver, where it is converted to urea and glutamine through the action of glutamine synthase [177]. However, in liver failure, ammonia bypasses the liver and reaches the brain due to portosystemic shunting [177, 178]. As a result, ammonia can reach other organs, including the brain, by crossing the BBB. In the brain, astrocytes are involved in ammonia metabolism, but increased production of glutamate leads to osmotic stress and swelling, resulting in brain edema [179–181]. Furthermore, in the presence of liver cirrhosis, gut dysbiosis occurs, leading to neuronal inflammation and intoxication. Astrocyte swelling further impairs neurotransmission and GABAergic activity. Additionally, bacterial translocation can lead to microglial activation and release of inflammatory cytokines, causing oxidative stress [129, 182].

Pathological dysregulation occurs in the small intestine, resulting in small intestinal bacterial overgrowth and a shift in bacterial diversity towards gram negative bacteria. The intestinal microbiota changes from nonpathogenic bacteria such as *Bacteroidetes*, *Ruminococcus*, *Roseburia*, *Veillonellaceae*, and *Lachnospiraceae* to pathogenic bacteria including overgrowth of *Fusobacteria*, *Proteobacteria*, *Enterococcaceae*, and *Streptococcaceae* [128, 129]. This leads to a decrease in the abundance of bacteria that produce SCFAs and disruption of gut barrier integrity. Consequently, the increased presence of bacterial products such as endotoxins in the gut can cause heightened inflammation and neuroinflammation [130, 131]. Therefore, there is a clear connection between the gut, liver, and brain in the pathogenesis of HE.

The GLB axis is a complex system involving various mechanical and biochemical processes that regulate overall health. Dysfunction in any of these processes can contribute to disease, potentially leading to severe or even fatal outcomes. Given the gut's central role in maintaining homeostasis and immunity, it presents a promising therapeutic target for disease management. Since gut microbiota and their metabolites play a crucial role in sustaining this axis, further exploration of their potential therapeutic applications is warranted.

## 6. Therapeutic Intervention

To overcome the disease progression, several abs or other medication is prescribed, but one way or in another way they also cause side effect and responsible for gut dysbiosis. As the gut dysbiosis plays a key role in disease development, therapeutic invasion should be involving them or their byproducts. As the gut dysbiosis take place it would eventually increase the rate of infection. From last two decades therapeutics involving the gut microbiota comes in research. Even the byproduct and metabolites are also used for the protective activity.

### 6.1. Probiotics

There are various probiotic species which are well known for their protective activity. Majorly *Lactobacillus sp*. is well known for their protective activity and highly in research. There are clinical trials going on for MAFLD with *Lactobacillus sp*. which showed the decreased level of steatohepatitis and change in ALT/AST profile. It also affected the blood insulin level and help in maintaining gut eubiosis. Further, *Lactobacillus rhamnosus* GG showed protective effect by modulating the inflammation and repairing the gut barrier integrity in AALD zebrafish model [183]. Even a group of researchers found that *Lactobacillus sp*. act as an antagonist for IL1 receptor and showed protective effect by balancing the Treg cell and Th17 cells in intestinal barrier [184, 185]. Not only *Lactobacillus*, but *A. muciniphila*, a novel organism, showed a positive response against type 2 diabetes and obesity related disorders. Not only the bacteria itself but its byproduct and extracellular vesicles tend to show protective activity by enhancing the β oxidation and elevating the mucosal barrier [186–189]. Even for IBD there are various research went on with *Lactobacillus*, *Faecalibacterium prausnitzii*, and *Bifidobacterium longum* by increasing the anti-inflammatory response and inhibiting the NF-κB activity [190–193]. Soliman et al. [194] studied the protective effect of *Lactobacillus acidophilus* and *Bifidobacterium lactis* along with amoxicillin showed anti-inflammatory and increased immunomodulatory activity in acute diverticulitis disease. Further, for AALD *Bifidobacterium animalis* subsp. *lactis* KV9 showed mitigative effect by suppressing TNF-α [195].

### 6.2. Fecal Microbiota Transfer (FMT)

To restore the microbial balance in diseased person, administration of microbial community from a healthy donor stool is known as FMT. It is mentioned by Nigam et al. [196] in their review that FMT is a more successful method than administration of abs for intestinal disease. There are few criteria that should be followed to become a FMT donor. A donor should have healthy life style without chronic history and family diseases [197]. There are few studies done for ulcerative colitis (UC) patient which showed that after 4 weeks of the FMT transfer patient showed the significant change in gut microbiota and the change was almost similar to healthy donor [198]. Furthermore, who are nonresponsive to mesalazine or prednisone in the long-term showed positive response for FMT by downregulating pro-inflammatory cytokines and inflammation [199]. Further there are other few studies that indicate the positive activity of FMT in HE with increased diversity of beneficial taxa [128, 200]. Insulin resistant patient with improved intestinal health and improved γ-aminobutyric acid and improved insulin sensitivity [201, 202]. Along to that a study on mice model showed that mice with FMT showed intestinal microbiota closely similar to resistant donor mice which further prevented steatosis, liver inflammation, and restored gut homeostasis [203, 204]. Although FMT is recognized as a promising therapeutic approach, two clinical trials were conducted in phases 1 and 2 for MAFLD; however, their results were not available. Instead, numerous randomized preclinical trials are currently underway, as listed in Tables 1 and 2. Even with that there is one clinical trial conducted by Indian investigator from Institute of Liver and Biliary Sciences, India, showed after FMT, reduction in hepatic and systemic inflammatory markers like TNF-α, c reactive proteins, and serum endotoxins were reduced.

### 6.3. Postbiotics

Postbiotics are defined as “preparation of inanimate microorganisms and/or their components that confers a health benefit on the host” [227]. This term is well known from last approx 15 years. They are also known as ”ghost probiotics,” ”paraprobiotics,” ”parapsycobiotics,” ”tyndallized probiotics,” ”metabiotics,” and ”bacterial lysates [228–237]. They are the cell metabolites majorly are SCFAs like butyrate, propionate, and acetate. Along with that some endotoxin, vitamins, and bacteriocin are known postbiotic substances. Among all of them, SCFAs are majorly in research due to their activity. In MAFLD, activity of butyrate, acetate, and propionate is studied well and showed the protective response by significantly decreasing the pro-inflammatory cytokine level and eventually maintain the barrier integrity [115, 238–240]. Even there are some studies that shows the effect of SCFAs on HE which still needs proper explanation and understanding [241]. While for IBD, SCFAs shows protective effect by maintaining the barrier integrity and anti-inflammatory activity [242].

Butyrate, in particular, promotes anti-inflammatory activity against LPS inflammation and promote polarization of M2 macrophage and inhibit the M1 macrophage activity [243]. Furthermore, butyrate also might show anti-inflammatory activity via activating GPR109A and inhibit M1 macrophage polarization through HDAC activity [244, 245].

### 6.4. Prebiotics and Synbiotics

According to International Scientific Association for Probiotics and Prebiotics (ISAPP) defined “dietary prebiotics” as “a selectively fermented ingredient that results in specific changes in the composition and/or activity of the gastrointestinal microbiota, thus conferring benefit(s) upon host health” [246]. There are different kind of prebiotics are there like fructans, galacto-oligosaccharide, starch, and glucose-derived oligosaccharides and noncarbohydrate oligosaccharides. Naturally prebiotics are the different dietary food which include garlic, beet, banana, barley, tomato, rye, soybean, human's and cow's milk, peas, beans, et cetera, and recently, seaweeds, microalgae, and onion [247]. There are few studies that shows the protective effect of prebiotic material in amelioration of MAFLD by increasing the SCFA production and decreases the ration of firmicutes and bacteroidetes like *Ganoderma lucidum*, *Hirsutella sinensis*, *Polygonum multiflorum*, and *Bupleurum Radix*. These all are Chinese herbs and well known for their activity against MAFLD [248]. Even recently, Do et al. [249] showed the protective effect of Gellan gum against MAFLD by modulating gut microbiota and metabolites. It also upregulated SCFA production and inhibited the inflammatory cytokine response.

Synbiotics are the mixture of prebiotics and probiotics. A prebiotic material is utilized by probiotics for their own growth. It is a synergistic effect, where substrate is selectively utilized by coadministered organism. Even the combination also helps in maintaining the intestinal mucosa [250]. Recently, fructose, xylose oligosaccharide, and stachyose: resistant dextrin was used in combination as prebiotic and along with that *L. acidophilus*, *Bifidobacterium lactis Lactobacillus reuteri*, *L. rhamnosus*, and *Streptococcus thermophilus* were used in mixture with prebiotic of 1:1 ratio, showed anti-inflammatory effect, and strengthen the intestinal barrier in acute colitis mice [251]. Even the tyrosol extracted from olive oil with combination with *Lactobacillus plantarum SC-5* replenish normal physiological bacteria and inhibit harmful intestinal bacteria, which can alleviate the symptoms of UC [252]. Patel et al. [218] showed the protective effect of aged garlic extract and *L. rhamnosus* MTCC1423 for AALD rat model. In colon, synbiotic reduced the activity of pro-inflammatory cytokine, while increased the barrier integrity by upregulation of ZO-1 and occludin.

While gut microbiota-based therapies show promise, several research gaps remain. Postbiotics have been extensively studied in the gut–brain axis, but the roles of probiotics and prebiotics in this context are less explored, despite substantial data on the gut–liver axis. SCFAs dominate postbiotic research, yet BAs, lactate, and succinate remain underexplored. FMT studies for MAFLD are ongoing, but its effects on AALD require further investigation. The crucial roles of microbial metabolites and microbes necessitate rigorous in vivo studies, as outlined in Table 1. Additionally, research on probiotics, prebiotics, FMT, and postbiotics for fatty liver disease is largely centered on MAFLD, with limited publicly available data. Some randomized controlled trials on MAFLD and AALD have shown positive effects of synbiotics, probiotics, and prebiotics, as summarized in Table 2.

## 7. Conclusion

The GLB axis is a complex and bidirectional communication network that regulates metabolic, immune, and neurological functions. Gut microbiota and their metabolites play a crucial role in maintaining the integrity of these three major organs. Key metabolites such as SCFAs and BAs help sustain gut homeostasis, modulate immunity, and provide protection to both the liver and brain. They reduce inflammation by promoting anti-inflammatory responses and help maintain the integrity of tight junctions in the BBB and gut barrier. Disruptions in gut microbiota or their metabolites can contribute to liver diseases such as MAFLD and AALD, as well as neuroinflammatory disorders like schizophrenia and HE. The translocation of microbial metabolites and endotoxins from the gut to the liver and brain triggers immune activation, oxidative stress, and inflammatory responses, further accelerating disease progression.

Given the vital role of gut microbes and their metabolites in both disease progression and prevention, microbiome-based therapies hold significant potential for managing fatty liver disease and neuroinflammatory disorders. Probiotics, prebiotics, synbiotics, postbiotics, and FMT are promising therapeutic strategies aimed at restoring microbial balance, enhancing intestinal barrier function, and reducing systemic inflammation. However, further research is essential to refine these interventions and develop personalized microbiome-targeted therapies for liver and neurological disorders. A deeper understanding of the GLB axis will be crucial in advancing novel and effective treatments for metabolic and neuropsychiatric diseases.

## Figures and Tables

**Table 1:** Randomized clinical trails for FMT, probiotic, prebiotic, postbiotic, and symbiotic on liver and brain disease.

Disease type	Treatment type	No. of days of the study and patient	Outcome of the study	Reference
MAFLD	Synbiotic (fructo-oligosaccharides and *Bifidobacterium animalis* subsp. *lactis* BB-12 at a minimum of 10 billion CFU/day)	102 patient with 12 month study	Changed the gut microbiota but liver fat change was not seen. Increased *Bifidobacterium* and *Faecalibacterium*.	[205]

MAFLD	Probiotic (VSL#3)	35 patient for 10 weeks	No significant change in liver marker.	[206]

MAFLD	Probiotic	39 patient (19 in NASH while 20 as placebo control) for 1 year	Significant improvement in ALT and AST, leptin, TNF-α, and endotoxin.	[207]

MAFLD	FMT	Total 72 cases where FMT *n* = 47 and non-FMT group *n* = 28 a 4 week study, where patients were administered to FMT by colonic infusion per 1 day	Microbial change to healthy individual after FMT, change in fat accumulation in liver.	[208]

MAFLD	Postbiotic (butyrate based formula)	50 participants with 12 week intervention period	Improved fatty liver index and change in GGT level and TG level.	[209]

MASH	Supplement of probiotic mix *Lactobacillus acidophilus* (1 × 10^9^ CFU) + *Lactobacillus rhamnosus* (1 × 10^9^ CFU) + *Lactobacillus paracasei* (1 × 10^9^ CFU) + *Bifidobacterium lactis* (1 × 10^9^ CFU)	45 patients with 24 weeks study	CK-18 was reduced in placebo and treatment group, with a larger effect of probiotic group. TLR4 was also reduced equally in both the groups.	[210]

Alcoholic hepatitis	Probiotic supplementation	100 patients with 7 days treatment	Altered gut microbiota in patients with disease, reduced endotoxins and alcohol induced gut dysbiosis.	[211]

AALD	Probiotic strain *Lactobacillus casei*	158 patients with 60 days treatment	Improvement in lipid metabolism and regulated overall intestinal flora.	[212]

AALD	Probiotic beverage Yakult 400 (Y400), which contains *Lactobacillus casei* strain Shirota	37 patients with 4 weeks study	Reduced c-reactive proteins but not interleukin-6, corrected imbalance of gut microflora in treatment group.	[213]

Sever alcoholic hepatitis (SAH)	FMT	13 patients given FMT and 20 patient given standard care for 90 days	Patient with FMT care were found significantly better than standard care patient with improvement in HE and decreased IL-1β.	[214]

Schizophrenia	Probiotic and Vitamin D3 (50,000 IU vitamin D3 every 2 weeks plus 8 × 10^9^ CFU/day mix probiotic strain)	60 patients and (50,000 IU vitamin D3 every 2 weeks plus 8 × 10^9^ CFU/day mix probiotic strain) [Trial Reg. IRCT2017072333551N2]	Increased total antioxidant capacity and decreased malondialdehyde.	[215]

**Table 2:** Shows the therapeutic agent activity on different liver related disease.

Sr. no.	Name of therapeutic agent	Type of therapy	Disease condition	Result	Reference
1	Symbiter (14 alive probiotic strains of *Lactobacillus* + *Lactococcus* (6 × 1010 CFU/g), *Bifidobacterium* (1 × 1010/g), *Propionibacterium* (3 × 1010/g), *Acetobacter* (1 × 106/g) genera)	Probiotic	MAFLD	Modulated gut microbiota, reduced liver fat and serum AST and GGT in treated group, also TNF-α and IL-6 levels changed.	[216]

2	Cotreatment with probiotic V and Metformin	Probiotic	AALD	Cotreatment with probiotic V and Met improved ethanol-induced mucosal barrier dysfunction, oxidative stress, and TJ disruption. It enhanced TJ protein expression, reduced ER stress, and inhibited CYP2E1 and NOX gene expression. Additionally, it increased Nrf-2 translocation and antioxidant gene expression, reducing ROS and malondialdehyde levels.	[132]

3	*Bifidobacterium animalis* subsp. lactis V9 (V9)	Probiotic	MAFLD	V9 alleviated HFD-induced hepatic steatosis by reducing ALT, AST, triglycerides, and inflammation while restoring AMPK, PPAR-α, and glycogen levels. It downregulated SREBP-1 c, FAS, and inflammatory mediators (IL-6, IL-1β, TNF-α, TLR4, TLR9, NLRP3, ASC) and suppressed ERK, JNK, AKT, and NF-κB activation.	[217]

4	Aged garlic extract and *Lactobacillus rhamnosus*	Synbiotic	AALD	The synbiotic inhibited CYP2E1 activation and inflammatory markers (TNF-α, IL-6) while increasing ZO-1, occludin, and IL-10 expression. It promoted Lactobacillus colonization, restoring barrier function, microbiota balance, and reducing colon oxidative stress.	[218]

5	Combination of probiotics (*Streptococcus*, *Bifidobacterium* and *Streptococcus thermophilus*) and prebiotics (Inulin)	Synbiotic	MAFLD	The combination improved lipid metabolism, insulin resistance, and inflammation by activating AMPK and NF-κB. It restored gut barrier function, reduced the firmicutes/bacteroidetes ratio, and modulated the gut-liver axis, highlighting its potential for NAFLD prevention.	[219]

6	Formula 3 (mixture of resistant starch, fructooligosaccharide, inulin and xylooligosaccharide)	Synbiotic	MAFLD	Prebiotics reduced hepatic steatosis and cholesterol without weight loss, with optimal metabolic benefits when intake was limited to the active phase. They altered the gut microbiota, increased SCFA production, and promoted bacterial groups linked to improved metabolic health.	[220]

7	β-Glucan	Prebiotic	MAFLD	β-Glucan supplementation reduced adiposity, weight gain, and improved glucose tolerance by increasing energy expenditure and activity. It uniquely raised cecal butyrate levels, while all fibers shifted gut microbiota, short-chain fatty acids, and bile acid composition.	[221]

8	*Lactobacillus plantarum*-derived postbiotics (LP-cs)	Prebiotic	AALD	LP-cs improved alcohol-induced liver injury by enhancing cell survival, restoring AST, ALT, SOD, TC, and TG levels, and modulating lipid metabolism genes. It increased gut microbiota, especially *Akkermansia muciniphila*, and protected hepatocytes from oxidative damage.	[222]

9	Heat-killed *Lactobacillus johnsonii* (HKLJ)	Postbiotic	AALD	HKLJ enhanced intestinal lysozyme expression, activating the NOD2-IL-23-IL-22 axis to maintain gut homeostasis and stimulate STAT3 for liver repair. It corrected microbiota dysbiosis by restoring butyrate-producing bacteria and reducing opportunistic pathogens.	[223]

10	10 mL/kg of FMT bacterial solution from donor rats	FMT	AALD	FMT improved liver function, survival, and intestinal barrier, while reducing inflammation and liver fibrosis in injury models. It also corrected gut dysbiosis and enhanced metabolic pathways in AALD rats.	[224]

11	FMT with human donor stool (heterologous) via colonoscopy	FMT	MAFLD	FMT reduced hepatic fat accumulation by improving gut microbiota dysbiosis, thereby alleviating fatty liver disease. Significant differences in clinical features and gut microbiota were observed between lean and obese MAFLD patients. Additionally, FMT was more effective in restoring gut microbiota in lean MAFLD patients than in obese ones.	[208]

12	Obeticholic acid (OCA)	FXR agonist	MAFLD	Significantly improved liver biochemistry and histology in patients with MASH.	[225]

13	Glucosamine (GLC)	Amino monosaccharide	MAFLD	GLC improved insulin resistance, inflammation, and antioxidant function while regulating serum and liver lipid metabolism. It enhanced intestinal barrier function, reduced LPS translocation, and inhibited the LPS/TLR4/NF-κB pathway, thereby alleviating liver inflammation and MAFLD-related liver damage.	[226]

## Data Availability

All data presented in this review are from previously published studies and are cited accordingly; no new data were generated.
